# Effects of sEA on Slow Transit Constipation through the Microbiota-Gut-Brain Axis in Rats

**DOI:** 10.1155/2020/8828846

**Published:** 2020-12-14

**Authors:** Xun Jin, Yanting Guan, Hua Bai, Yan Liu, Xing Lv

**Affiliations:** ^1^Nanjing University of Chinese Medicine, 138 Xianlin Avenue, Qixia District, Nanjing 210023, China; ^2^Oncology Business Development Department, Hutchison MediPharma Limited, Shanghai 201203, Building 7, 898 Halei Road Zhangjiang Hi-Tech Park Pudong, China

## Abstract

To investigate the effect of sacral electroacupuncture (sEA) on the microbiota-gut-brain axis in the treatment of slow transit constipation, this study established a drug-induced model of slow transit constipation in rats and carried out sEA at the Baliao acupoints (BL31-BL34). On the 14th day of the therapeutic period (24 h fecal pellets), the aquaporin 3 (AQP3), 5-hydroxytryptamine (5-HT), and substance *P* (SP) transcripts from the distal colon and hypothalamus were analyzed. 16S rDNA has been widely used to analyze the diversity of the microbial communities. Therefore, in the present study, changes in the intestinal microbiota were analyzed by 16S rDNA gene sequencing. The results showed that sEA significantly increased the number of fecal pellets and the water content in the feces and reduced the reabsorption of intestinal water in 24 h. sEA also upregulated the level of SP mRNA expression in the distal colon and the hypothalamus, but downregulated the level of 5-HT mRNA expression in the distal colon. Moreover, sEA improved the Bacteroidetes to Firmicutes (B/F) ratio, which is beneficial to the general structure of the intestinal microflora. Our findings suggested that the microbiota-gut-brain axis constitutes a crucial pathological basis in the development of slow transit constipation. sEA improved the slow transit constipation by regulating the balance of the microbiota-gut-brain axis.

## 1. Introduction

Functional constipation (FC) is an idiopathic intestinal disease and with an increasing prevalence rate, year by year [1]. FC's morbidity rate ranged from 1.9% to 40.1% with an average of 14% [2, 3]. The clinical self-test report rate of FC is generally higher than that obtained from the Rome Diagnostic Criteria, a guideline for the diagnosis and treatment of functional gastrointestinal disorders. According to the Rome IV Criteria, FC is classified into three types: (1) normal transit constipation, (2) slow transit constipation (STC), and (3) defecation difficulty or rectum defecation difficulty. These three different types of FC may combine and overlap with each other [1]. STC is characterized by decreased motility of the colon. Its main manifestations include decreased defecation frequency (<3 times/week), low desire for defecation, abdominal distention, and delayed colonic transit time (CTT) [1].

The disorder of gut-brain axis regulation is an essential pathological foundation of STC [1, 4]. The imbalance of intestinal microflora also contributes to the generation and development of STC. These factors (gut-brain axis and microbiota) may interact with each other [5–9]. Thus, we proposed that the microbiota-gut-brain axis is involved in STC pathological mechanisms. The axis consisted of the intestinal microflora, enteric nervous system (ENS), automatic nervous system (ANS), neuroendocrine-neural immune system, and central nervous system (CNS). These systems could regulate gastroenteric physiological functions and STC pathological processes [5, 8, 10]. The hypothalamic-pituitary-adrenal axis (HPA) is the pivot for the neuroendocrine immune network and one of the main communication networks between the colon and brain [11]. The “consensus opinion of Chinese chronic constipation experts” recommended the use of selective 5-HT4 receptor agonists and suggested that probiotics could be one of the treatment options for patients with FC [12]. Mosapride is widely used in the clinic and has an obvious effect on promoting gastrointestinal motility. *Bacillus licheniformis* is a commonly used probiotic with low incidence of adverse reactions [13–15]. Probiotics not only relieve STC gastroenteric symptoms but also improve anxiety and depression, which are associated with STC [8, 16–21]. Several findings demonstrated that the neural regulation of colon motility and the participation of brain-gut peptides played an important role in sacral neuromodulation [22, 23]. However, their relationship to intestinal microflora remains unclear.

Our previous studies have demonstrated that sEA applied to Baliao acupoints has a benefit for sacral neuromodulation [24, 25]. The Baliao acupoints are located symmetrically in the four pairs of posterior sacral foramina. Nerve impulses induced by the stimulation of the Baliao acupoints enter the S1–S4 spinal sections near the level of the sacral medullary defecation center (S2–S4) and then triggers the cortex-lumbar-anorectal nerve pathway to modulate effector organs and the colon [26–28]. A clinical study has shown that sEA can increase the frequency of defecation, relieve straining, improve the sensation of incomplete evacuation, promote defecation sensation, and eliminate abdominal distention. During treatment, patients felt their appetite, fatigue, sleep quality, and emotions improved [25, 26]. 16S rDNA has been widely used to analyze the diversity of the microbial community. Therefore, we used 16S rDNA to observe the impact of sEA on the intestinal microecological environment. Therefore, we sought to determine the role of sEA in STC through the microbiota-gut-brain axis in a well-accepted model of STC induced by loperamide in rats.

## 2. Materials and Methods

### 2.1. Animal Experiments

All animal procedures were performed following ethical principles in animal research and approved by the Nanjing University of Chinese Medicine. A total of 60, 12-week-old Sprague–Dawley (SD) rats (both gender) with bodyweights of 220–250 g were purchased from Shanghai Super-B&K Laboratory, Shanghai, China. Animals were kept in Central Animal House, Xianlin Campus, the Nanjing University of Chinese Medicine. They were housed at an ambient temperature of 22 ± 2°C and relative humidity of 50–70%, maintained under a normal 12-hour light/dark cycle and allowed access to food and water ad libitum.

**Figure 1:**
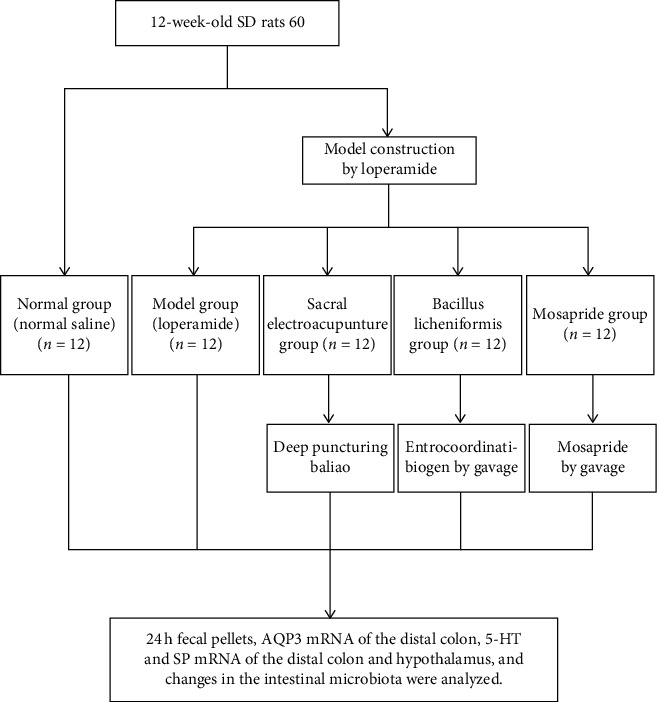
Flow diagram.

After two weeks of acclimatization, the rats were randomly assigned to following 5 groups (*n* = 12 per group; Figure 1): the normal group in which normal saline was administered, STC model group, sEA group, *Bacillus licheniformis* group in which intestinal microflora was managed, and mosapride group in which the gastrointestinal tract and motility were stimulated by an activator of the 5-HT4 receptor. The STC model was established in all groups except for the normal group. Loperamide was used to establish the STC rat model [29, 30]. The suspension of loperamide (produced by Xian Janssen Pharmaceuticals Company, bought from Zhilin Pharmacy, Nanjing, China with OTC No. H10910085) was administered as 2 ml of 3 mg/kg/d by IG, 1/d for 14 d consecutively.

On the 14th day of the therapeutic period, 24 h fecal grains and aquaporin 3 (AQP3), 5-HT, and SP mRNA of the distal colon and hypothalamus were examined, and changes in the intestinal microbiota were analyzed by 16S rDNA gene sequencing (Figure 1).

### 2.2. sEA Application

sEA was performed in the rat in the sEA group. Rats have only three pairs of posterior sacral foramina. Their 2nd and 3rd pairs are similar to those of humans: Ci Liao (BL 32), Zhong Liao (BL 33), and Xia Liao (BL 34) [31]. The root section of the tail of the rat was repeatedly pinched and relaxed to stimulate the active joint, which is the junction of the 1st coccyx and the sacral bone. Superiorly, two spinal processes from the junction are skipped to arrive at the intervertebral space between the 2nd and 3rd sacral spinal processes. The Ci Liao (BL 32) and Zhong Liao (BL 33) are located 5–10 mm lateral to the slightly superior border of the 2nd and 3rd intervertebral space. The Xia Liao (BL 34) is located 5–10 mm lateral to the slightly superior border of the 3rd and 4th intervertebral space. The acupuncture needle (0.35*∗*25 mm filiform, produced by Suzhou Medical Sino-foreign Joint Venture Suzhou Hua Tuo Medical Instruments Co., Ltd., Suzhou, China) was inserted approximately 15–20 mm with penetration of the skin through the 2nd and 3rd posterior sacral foramina to touch the anterior branch of the 2nd and 3rd nerve roots [32, 33]. Electroacupuncture apparatus (Hua Tuo SDZ-II, produced by Suzhou Medical Sino-foreign Joint Venture Suzhou Hua Tuo Medical Instruments Co., Ltd., Suzhou, China) was connected to two pairs of electrodes that were attached bilaterally to needles placed at 2nd and 3rd pairs of posterior sacral foramina in rats, which mimic Ci Liao (BL 32)/Zhong Liao (BL 33) and Xia Liao (BL 34) of humans. sEA was performed with a frequency of 2–15 Hz and a rarefaction-dense wave, lasting for 30 min. The intensity of electric current (1–1.5 mA) was controlled as interior rotation or contraction of the rat's thigh was noted. sEA treatment was performed daily for 14 days.

### 2.3. Medical Intervention

Normal saline (2 ml) was administered once a day by IG in the normal group.

Entrocoordinatibiogen was administered in rats in the *Bacillus licheniformis* group. A suspension of entrocoordinatibiogen (Shenyang No. 1 Pharmaceuticals Plant, bought from Zhilin Pharmacy, Nanjing, China with OTC No. S10950019) was administered as 2 ml of 1 billion viable bacteria/kg/d by intragastrical administration (IG), once a day for 14 consecutive days.

Mosapride was taken in rats in the mosapride group. A suspension of mosapride (produced by Jiangsu Hansoh Pharmaceutical Group Co., Ltd., bought from Zhilin Pharmacy, Nanjing, China with OTC No. H19990315) was administered as 2 ml of 3 mg/kg/d by IG, once a day for 14 days.

### 2.4. Measuring the Number of 24 h Fecal Pellets and Fecal Water Content

The feces passed for 24 h in each group were collected, and the number of pellets was recorded.

Fresh feces of rats were collected for 3 hours. The wet weight of feces was weighed first, dried the feces in a constant temperature drying oven, and then, the dry weight of feces was weighed. The percentage of fecal water content = (wet weight − dry weight)/wet weight × 100%.

### 2.5. Testing the Relative mRNA Expression of AQP3, 5-HT, and SP Using qRT-PCR

We took a total of 150 mg fresh segment of the distal colon, two cm away from the anus to examine mRNA expression of 5-HT, SP and AQP3. Additionally, the rats' skulls were broken to remove the brain. The entire hypothalamus was harvested to test the mRNA expression of 5-HT, SP, and AQP3. The hypothalamic tissue was placed into a sterilized EP tube (1.5 ml) and stored in an ultralow temperature freezer at −80°C. The tissue samples of colon and hypothalamus were processed to assess mRNA expressions of 5-HT, SP, and AQP3 using qRT-PCR. The RNA extraction kit (Invitrogen, California, US) was used to extract genetic RNA. The RNA evaluation was conducted by ultraviolet radiation or visible range spectrophotometer (provided by Hangzhou Allsheng Instruments Co., Ltd, Hangzhou, China/Nano100), and agarose gel electrophoresis was used to test the quantity, purity, and quality of the finally received RNA. Afterward, the RNA was reversely transcribed into cDNA by using a reverse transcription kit (Invitrogen, California, US), which was prepared for the subsequent gene testing. A pair of primers was designed by using the primer design site (http://sg.idtdna.com/Primerquest/Home/Index) and purchased from Hangzhou Qin Ke Zi Xi Biotechnology Co., Ltd. The primer code sequences were as follows: 5-HT: forward 5′-AGGACCAGAGCCACAATGAAA-3′, reverse 5′-CGTGAAAGGAAGACGGTGAAG-3′; SP: 5′-forward CAGAGGGCAGCACTTAGTTTA-3′, reverse 5′-TGAGCGTTCATTCAAGGTAGC-3′; and AQP3: forward 5′-GCTGCTGTGCCTATGAACTGA-3′, reverse 5′-CTTCTTGGGTGCTGGGATTGT-3′. The qPCR fluorescence quantitation kit (Applied Biosystems, California, US) was chosen, and cDNA template and real-time fluorescence quota PCR instrument (CFX384 TouchTM Real-Time PCR Detection System, Bio-Rad Co., California, US) were used to test the 5-HT, SP, and AQP3 genes. The reaction was performed in a 10 *µ*l system (5 *µ*l, 0.2 *µ*l, 0.2 *µ*l, 1 *µ*l, 0.2 *µ*l, and 3.4 *µ*l of 2 × ChamQ SYBR qPCR Master Mix, forward primer 10 *µ*M, reverse primer 10 *µ*M, template DNA, 50 × ROX Reference Dye 1, and nuclease-free H_2_O, respectively). The program was set to two steps for real-time quantitation: initial denaturation was performed at 95°C for 10 min. Subsequently, each denaturation was 95°C for 15 s, followed by an annealing elongation at 63°C for 30 s. The above steps composed of one cycle, and there were 40 cycles in total. The fluorescence value was read during each extension stage, and the dissolution curve was prepared after the end of the cycle. Each sample was performed in triplet, and ultimately, the relative expression levels of each gene were analyzed with the 2^−∆∆Ct^ method.

### 2.6. Extraction of Fecal DNA and Sequencing of 16S rDNA Gene

A sample of fresh feces (approximately 200 mg, 6∼8 grains) was collected and placed in an aseptic centrifuge tube (2 ml). The centrifuge tube was transported to the laboratory in a dry-ice box, and DNA extraction was immediately carried out. The genomic DNA of fecal bacteria was extracted using a DNA extraction kit (Stool DNA Kit 200, OMEGA Bio-Tek, Georgia, US). The Phusion enzyme amplified the corresponding high variable region, and 25∼35 cycles of amplification were performed with 50 ng of DNA template in a 25 *µ*L PCR system. Since the lengths of different species were slightly different, primers were designed in the target region around the peripheral conserved areas of V3 and V4 and approximately 468 bp in length. After one round of amplification, different adapters and barcodes were added to both ends of the forward and reverse primers and then amplified further. The amplified PCR products were purified on AMPure XT beads (Beckman, Coulter Genomics, Danvers, MA, US) and sequenced after a quantitative analysis of Qubit (Invitrogen, California, US). We analyzed the sequencing data using Illumina MiSeq 2 × 300 bp paired-end reads. For the dual-ended data obtained by MiSeq sequencing, first we divide the samples according to the barcode information, and then, we divide the combination into labels using the overlap relationship. We then filtered the data and performed a quality control analysis of Q20 and Q30. The final clean data were obtained using OTU clustering and taxonomic analysis.

### 2.7. Data Analysis

The sequencing of all microbial groups was calculated by Verseach (v2.3.4), and clean tags with sequence similarity greater than 97% were selected as an OTU. The longest reads were selected as the representative sequence of the OTU, which was used for species classification annotation (RDP database, V 11.3). QIIME was used to compute (V 1.8.0) rarefaction estimates and the Shannon–Wiener index. Based on the results of each sample of the OTU, the distance between samples was calculated by weighted UniFrac algorithm, and principal coordinate analysis (PCoA) was completed.

### 2.8. Statistical Analysis

All data were expressed as the mean ± SD and analyzed using one-way ANOVA (SPSS 22 statistical software) to determine the differences between groups, with the least significant difference (LSD). Values were considered to be significantly different when *P* < 0.05.

## 3. Results

### 3.1. Effects of sEA on the Number of Feces in STC

**Figure 2:**
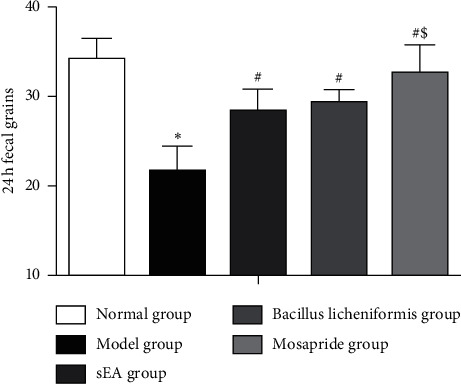
Changes in the number of 24 h fecal pellets in STC rats after sEA and medical treatment. ^*∗*^*P* < 0.05, comparison with the normal control; ^#^*P* < 0.05, comparison with the STC model; ^$^*P* < 0.05, comparison with sEA.

As shown in Figure 2, the number of 24 h fecal pellets in the STC model group was significantly lower than that in the normal group (*P* < 0.01). Compared to the STC model group, the number of 24 h fecal pellets in the sEA group was markedly increased (*P* < 0.05). The amount of 24 h fecal pellets was even higher in the mosapride group than that in the sEA group and the *Bacillus licheniformis* group (*P* < 0.05). There is no significant difference in the 24 h fecal pellets between the sEA group and the *Bacillus licheniformis* group.

### 3.2. The Change in Water Content in Feces in STC by sEA

**Figure 3:**
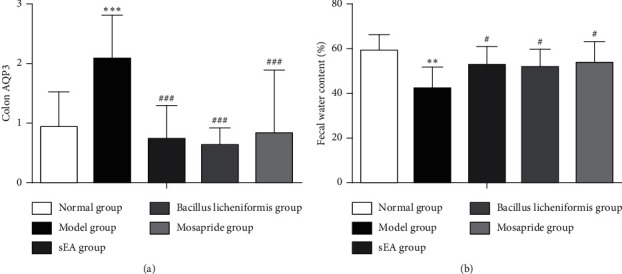
Changes in water content in feces after sEA and medical treatment. (a) The level of AQP3 mRNA expression in the distal colon. (b) The fecal water content. ^*∗*^^*∗*^^*∗*^*P* < 0.001, comparison with the normal control; ^###^*P* < 0.001, comparison with the STC model; ^*∗*^^*∗*^*P* < 0.01, comparison with the normal control; ^#^*P* < 0.05, comparison with the STC model.

Aquaporins (AQPs) is a group of small hydrophobic molecules with a high expression on the surface of the colon that is directly involved in water absorption and mucus secretion [33]. We observed that the AQP3 mRNA in the distal colon in the STC model group was significantly higher than that in the normal group, indicating that the water absorption in the distal colon was increased in STC. However, the AQP3 mRNA in STC rats treated with sEA, *Bacillus licheniformis*, or mosapride was markedly reduced (Figure 3(a)), compared to STC rats. There was no significant difference in water absorption in the distal colon among STC groups treated with sEA, *Bacillus licheniformis*, or mosapride.

Compared with the normal group, the fecal water content in the STC model group was significantly decreased (Figure 3(b)). Compared with the STC model group, the fecal water content was increased in sEA, *Bacillus licheniformis*, and mosapride groups (Figure 3(b)).

### 3.3. Alterations in mRNA Expressions of SP and 5-HT in the Distal Colon STC by sEA

**Figure 4:**
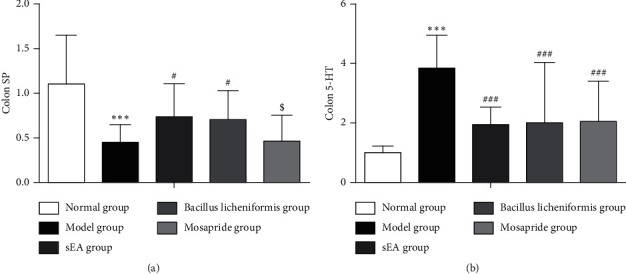
Alterations in mRNA expressions of SP and 5-HT in the distal colon by sEA and medical treatment. (a) The level of SP mRNA expression in the distal colon. (b) The 5-HT mRNA expression in the distal colon. ^*∗*^^*∗*^^*∗*^*P* < 0.001, comparison with the normal control; ^#^*P* < 0.05 and ^###^*P* < 0.001, comparison with the STC model; ^$^*P* < 0.05, comparison with sEA.

It was found that SP mRNA expression in the distal colon of STC rats treated with or without acupuncture or a medication (*Bacillus licheniformis* or mosapride) was significantly lower than that in normal controls (*P* < 0.05; Figure 4(a)). However, compared to STC without any treatment, sEA and administration of *Bacillus licheniformis* but not mosapride significantly increased SP mRNA expression in the STC distal colon. There was no difference in the SP mRNA expression between the sEA group and the *Bacillus licheniformis* group.

As demonstrated in Figure 4(b), we observed that 5-HT mRNA expression in STC distal colon was significantly elevated than that in normal controls. sEA, as well as intake of *Bacillus licheniformis* or mosapride, significantly decreased 5-HT mRNA expression in the STC distal colon (*P* < 0.05).

### 3.4. Influence of sEA on SP and 5-HT in the Hypothalamus

**Figure 5:**
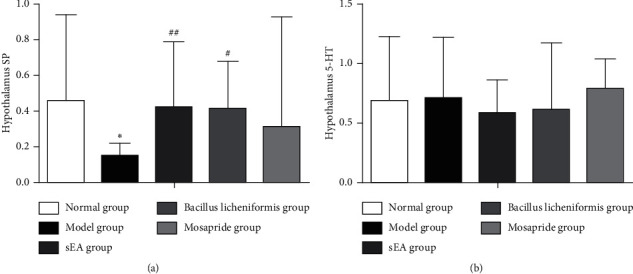
Influence of sEA and medical treatment on mRNA expressions of SP and 5-HT in the hypothalamus. (a) The levels SP of SP mRNA expression in the hypothalamus. (b) The 5-HT mRNA expression in the hypothalamus. ^*∗*^*P* < 0.05, comparison with the normal control; ^#^*P* < 0.05 and ^##^*P* < 0.01, comparison with the STC model.

We observed that SP mRNA expression in the hypothalamus of rats in the STC model group was significantly lower than that in the normal group (*P* < 0.05; Figure 5(a)). Compared to the STC model group, SP mRNA expression was markedly increased in the sEA group (*P* < 0.01) and *Bacillus licheniformis* group (*P* < 0.05), but not in the mosapride group. It was noted that there was no significant difference in 5-HT mRNA expression in the hypothalamus among all groups (Figure 5(b)).

### 3.5. Effect of sEA on the Diversity of Intestinal Microflora

**Figure 6:**
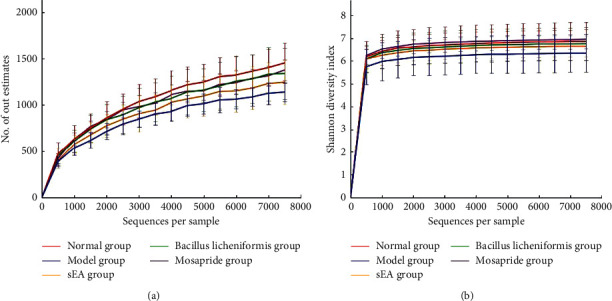
Analysis of intestinal microflora. (a) The rarefaction curves show the relation of the number of OTUs to the sequence number per sample in each group. (b) The Shannon curves indicate potential species of intestinal flora in each group.

In this study, a total of 1,318,519 high-quality sequences were obtained from 60 samples. There was an average of 21,975 sequences per sample. A total of 4,213 OTUs were obtained at 97% similarity with a proportion of 70 OTUs per sample. The sparse curve showed that the number of OTUs increased sharply when the sequence number was less than 4,000, and the increased number of OTUs tended to be slow as the sequence number increased to 7,000 (Figure 6(a)). The Shannon index of all the samples with saturations showed that this experiment had reflected most species of intestinal microflora and diversity of rats (Figure 6(b)).

**Figure 7:**
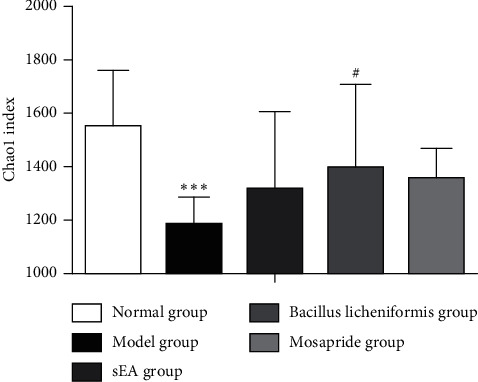
Changes in the Chao1 index of gut microbiota induced by acupuncture and medical treatments. ^*∗*^^*∗*^^*∗*^*P* < 0.001, comparison with the normal control; ^#^*P* < 0.05, comparison with the STC model.

The Chao1 index is used to estimate the total number of species of intestinal flora. The larger values of the Chao1 index correspond to the larger numbers of gut microbiota [16]. We observed that the Chao1 index in the model group was significantly lower than that in the normal group (*P* < 0.05; Figure 7). sEA and administration of the mosapride did not induce significant changes in the Chao1 index in STC rats, compared to the rat in the model group. In contrast, the administration of the *Bacillus licheniformis* significantly increases the Chao1 index in STC rats (*P* < 0.05).

### 3.6. Alteration in the Overall Structure of the Intestinal Microflora Induced by sEA

**Figure 8:**
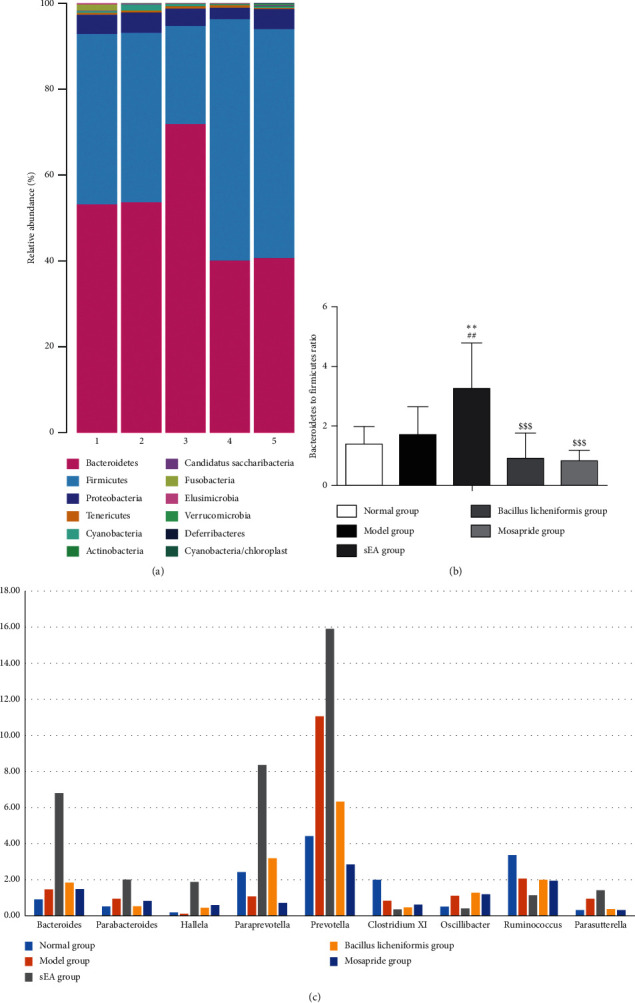
Changes in the overall structure of the intestinal microflora by sEA and medical treatments. (a) The bar charts represent the average relative abundances of the bacterial phylum. (b) Changes in the ratio of the Bacteroidetes to Firmicutes (B/F). ^*∗*^^*∗*^*P* < 0.01, comparison with the normal control; ^##^*P* < 0.01, comparison with the STC model; ^$$$^*P* < 0.001, comparison with sEA. (c) The bar charts represent the average relative abundances of the bacterial genes.

The dominant intestinal bacteria in each group were Firmicutes, Bacteroidetes, and Proteobacteria (Figure 8(a)). There was no significant difference in the composition of the gut flora at the phylum level between normal control and STC model groups. Compared to normal and STC groups, the formation of the intestinal bacteria in STC rats treated with sEA was significantly altered. In this regard, we found that the abundance of Bacteroidetes increased, the abundance of Firmicutes decreased, and the ratio of the Bacteroidetes to Firmicutes (B/F) elevated significantly (*P* < 0.05; Figure 8(b)). Besides, the B/F ratio was markedly higher in the sEA group than that in the *Bacillus licheniformis* group and the mosapride group (*P* < 0.001; Figure 8(b)).

It was found that the main Bacteroidetes at the genus level in the intestine included *Bacteroides, Paraprevotella,* and *Prevotella* in each group. As shown in Figure 8(c), sEA induced changes in intestinal bacteria at the genus level, in comparison with all other groups. These alterations in the sEA group include an increase in the abundance of *Bacteroides, Parabacteroides, Hallella, Paraprevotella,* and *Prevotella* (all Bacteroidetes), a decrease in the abundance of *Clostridium XI, Oscillibacter*, and *Ruminococcus* in Firmicutes, and an elevation in the abundance of *Parasutterella* in Proteobacteria.

## 4. Discussion

**Figure 9:**
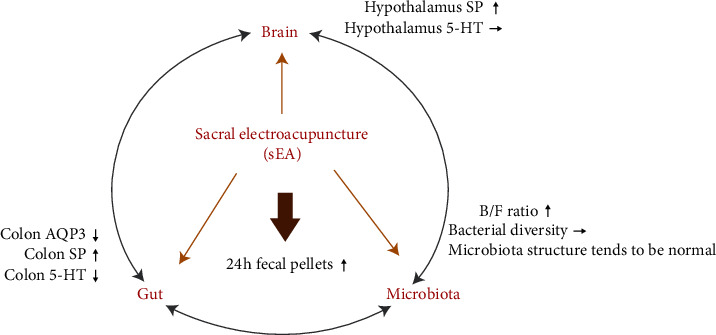
The schematic diagram shows the microbiota-gut-brain loop modulated by sEA in slow transit constipation in rats.

In the present study, we found that sEA exerted an overall regulatory effect on STC through the microbiota-gut-brain axis (Figure 9). Meanwhile, sEA significantly increased the number of 24 h fecal pellets and the water content in feces, but reduced the reabsorption of intestinal water. sEA could also improve the B/F ratio, leading to improve the general structure of the intestinal microflora. Moreover, it showed that sEA upregulated the SP mRNA expression in the distal colon and hypothalamus, but downregulated the 5-HT mRNA expression in the distal colon.

The efficacy of sEA for constipation is controversial. Previous studies have shown that different stimulation parameters of sEA may affect the curative effect [34–36]. Our previous clinical trials and other previously published reports showed that low-frequency (2 Hz–15 Hz) electroacupuncture was superior to high-frequency (100 Hz) for improving constipation [37, 38]. Therefore, in the present study, we used the low-frequency (2–15 Hz) as a stimulus parameter for sEA. We found that sEA significantly increased the fecal pellet and water content and accelerated the distal colon transit time in STC rats, suggesting the action of sEA was involved in improving constipation. Previous studies showed that sEA has no effect on gastric emptying and small intestinal transit time, but mainly affects the distal colon [38, 39]. Thus, others and our recent findings indicate that sEA might be beneficial for constipation in the distal colon.

Mosapride is a 5-HT4 receptor agonist that interacts directly with 5-HT4R of the intermuscular plexus and affects the total digestive tract power [40–42]. This interaction may be one reason why the 24 h fecal pellets in the STC rat treated with mosapride were significantly higher than those in the STC rat treated with sEA. The results suggested that the mosapride was better than sEA in promoting motility. We think that mosapride is a drug that can improve the entire gastrointestinal motility, while sEA mainly affects the colorectal portion and alleviates dysfunctions. With an excitatory effect on the ENS, an increased concentration of 5-HT could induce the abnormal function of colonic circular muscle, affecting gastrointestinal motility [43]. In contrast, 95% of 5-HT is generated in the gastrointestinal tract, while a small amount of 5-HT is produced in the brain. Since 5-HT cannot pass through the blood-brain barrier, the 5-HT exerts its effect separately in the ENS and CNS [44]. The ENS can independently regulate gastrointestinal function to a certain extent, even in the absence of external innervation [45]. Since there was a very low level of 5-HT existence in the hypothalamus, it may be a reason why there was no difference in the 5-HT mRNA expression in the hypothalamus among all experimental groups in the present study. Further research may require to address the changes in the hypothalamus in different courses of constipation treated with or without different types of acupuncture.

It was noted that sEA increased SP mRNA expression and decreased 5-HT mRNA expression in the distal colon. Meanwhile, sEA induced an elevation in SP mRNA in the hypothalamus. Our observations were consistent with other findings from clinical trials and animal experiments [46–51]. Interestingly, we found no change in the 5-HT mRNA expression in the hypothalamus of STC rats following sEA treatment. There is evidence showing that SP is mainly distributed in the hypothalamus. The level of SP is lower in the peripheral nerve system (PNS) than that in the central nerve system (CNS). The SP is widespread throughout the entire gastrointestinal tract. It can inhibit the secretion of gastric acid and stimulate intestinal motility [52], as one of the important factors that contribute to the initiation of the acupuncture action on the intestine [37, 39].

It was observed that sEA could transform the overall structure of STC rat flora toward a more normal composition by changing the B/F ratio. According to other previous reports [9, 53–59], at the phylum level, the proportion of Bacteroidetes in a healthy population is 58.18%, identified as the dominant flora, and the proportion of Firmicutes is 34.98%. The B/F ratio was inverse, representing a decrease in Bacteroidetes and an increase in Firmicutes, in a population with constipation. However, in the present study, we found that there was no significant decrease in the B/F ratio after model establishment, which may be related to the duration of constipation. After sEA treatment, at the phylum level, the abundance of Bacteroidetes was increased, while the abundance of Firmicutes was decreased in STC rats, respectively. Moreover, the B/F ratio in the STC rat treated with sEA was even higher than that in the normal controls without STC. Furthermore, at the genus level in STC rats, sEA markedly increased the profusion of *Bacteroides, Parabacteroides, Prevotella,* and *Paraprevotella*. The abundance of potentially pathogenic bacteria, such as *Clostridium* XI and *Ruminococcus*, was decreased compared to normal rats and STC rats treated with or without drugs, including *Bacillus licheniformis* and mosapride. Hence, our findings suggested that sEA has a beneficial effect on the intestinal microflora.

The diversity of bacteria is an abundance index of reactive species in intestinal microflora. The diversity loss in STC rats may decrease the flora stability and weaken the colonization resistance, resulting in an imbalance in the intestinal microflora [60]. Feces are an excellent medium for the intestinal microflora. In particular, hydrated feces are a suitable environment for microorganisms to compete and group together. Thus, the decrease in STC fecal water content also affects the diversity of intestinal microflora [16]. In the present study, we observed that there was no noticeable change in intestinal microflora diversity after sEA and the mosapride treatment, but a noticeable change after the treatment with *Bacillus licheniformis*. Our findings suggested that sEA may effectively change the composition of the flora, but may not improve the abundance of intestinal microflora species, as probiotics such as *Bacillus licheniformis* do. In this respect, we found that *Bacillus licheniformis* regulates intestinal function through the microbiota-gut-brain axis comparably to sEA. In addition to increasing bacterial diversity and improving 24 h fecal grains and water content in feces, which are consistent with other previous reports [16, 61, 62], *Bacillus licheniformis* upregulates SP mRNA expression in the distal colon and hypothalamus and downregulates 5-HT mRNA expression in the distal colon. Therefore, we propose that sEA and probiotics are complementary, which can affect different levels of the microbiota-gut-brain axis. It is possible to further explore the intervention time and dosage of probiotics during the treatment of STC with sEA to observe the synergistic mechanism between sEA and probiotics.

## 5. Conclusion

This study demonstrates that sEA can be used to manage STC by regulating the balance of the microbiota-gut-brain axis. However, clinical research has shown that the onset of the treatment of STC symptoms with sEA is different. Early improvements include increasing the defecation sensation, defecation frequency, and feeling of incomplete evacuation, as well as relieving abdominal distension. The cumulative effect of continued treatment leads to a reduction in the lumpy or hard stools and an increase in straining and defecation time [26]. During treatment, the STC recurrence mainly happened in patients with a general improvement [25]. Hence, the action of sEA can be classified into three periods: latency period, effect period, and posteffect period. There were significant difference in duration, intensity of impact, and direction of function in each period [63]. Therefore, it is necessary to further study the useful characteristics of sEA in terms of the bidirectional responsive mechanism in the microbiota-gut-brain axis to determine the time-effect curve of the treatment of STC with sEA.

## Data Availability

The datasets used and/or analyzed during the current study are available from the corresponding author upon request.

## Abbreviations

sEA: Sacral electroacupuncture
HPA: Hypothalamic-pituitary-adrenal axis
AQP3: Aquaporin 3
AQPs: Aquaporins
5-HT: 5-Hydroxytryptamine
SP: Substance *P*
B/F: Bacteroidetes to Firmicutes
FC: Functional constipation
STC: Slow transit constipation
CTT: Colonic transit time
ENS: Enteric nervous system
ANS: Automatic nervous system
CNS: Central nervous system
SD: Sprague–Dawley
BL 32: Ci Liao (BL 32)
BL 33: Zhong Liao (BL 33)
BL: Xia Liao (BL 34)
IG: Intragastrical administration (IG)
LSD: Least significant difference (LSD)
PNS: Peripheral nerve system (PNS).

## References

[1] Ke M., Fang X., Hou X. (2016). *Rome IV: Functional Gastrointestinal Disorders/Disorders of Gut-Brain Interaction*.

[2] Suares A. C., Ford A. C. (2011). Prevalence of, and risk factors for, chronic idiopathic constipation in the community: systematic review and meta-analysis. *American Journal of Gastroenterology*.

[3] Chang j. y., Locke g. r., Schleck c. d., zinsmeister a. r., talley n. j. (2007). Risk factors for chronic constipation and a possible role of analgesics. *Neurogastroenterology & Motility*.

[4] Hou-xun N., Sun J.-h. (2012). Pathogenesis of slow transit constipation. *Modern Journal of Integrated Traditional Chinese and Western Medicine*.

[5] Palma G. D., Collins S. M., Bercik P. (2014). The microbiota-gut-brain axis in functional gastrointestinal disorders. *Gut Microbes*.

[6] Tian H., Ding C., Gong J. (2016). Treatment of slow transit constipation with fecal microbiota transplantation. *Journal of Clinical Gastroenterology*.

[7] Tian H., Ge X., Nie Y. (2017). Fecal microbiota transplantation in patients with slow-transit constipation: a randomized, clinical trial. *PLoS One*.

[8] Ge X., Zhao W., Ding C. (2017). Potential role of fecal microbiota from patients with slow transit constipation in the regulation of gastrointestinal motility. *Scientific Reports*.

[9] Zhu L., Baker S. S., Alkhouri R. (2014). Structural changes in the gut microbiome of constipated patients. *Physiological Genomics*.

[10] Liu H.-n., Yu-zhuo C., Wu H., Tao-tao L. (2015). Research progress of functional constipation and intestinal microbiota. *Fudan University Journal of Medical Science*.

[11] Bernstein C. N. (2017). The brain-gut axis and stress in inflammatory bowel disease. *Gastroenterology Clinics of North America*.

[12] Gastrointestinal dynamics group (2019). Expert consensus on chronic constipation in China (2019, Guangzhou). *Chinese Journal of Digestive Diseases*.

[13] Yu-xue Y. (2020). Effect of probiotics combined with mosapride on constipation irritable bowel syndrome. *Chinese and Foreign Medical Research*.

[14] Ru-ru L. I. (2020). Effect observation of lactulose combined with *Bacillus licheniformis* capsules in the treatment of constipation-type irritable bowel syndrome. *China Journal of Clinical Rational Drug Use*.

[15] Jia-hu L. I. (2017). Clinical effect of *Bacillus licheniformis* combined with compound glutamine in the treatment of intestinal dysfunction. *Clinical Practice*.

[16] (2016). Consensus on clinical application of digestive tract microecological regulator in China. *Chinese Journal of Microecology*.

[17] Dimidi E., Whelan K., Fragkos K. C. (2014). The effect of probiotics on functional constipation in adults: a systematic review and meta-analysis of randomized controlled trials. *The American Journal of Clinical Nutrition*.

[18] Chmielewska A., Szajewska H. (2010). Systematic review of randomised controlled trials: probiotics for functional constipation. *World Journal of Gastroenterology*.

[19] Dimidi E., Whelan K., Scott S. M. (2017). Mechanisms of action of probiotics and the gastrointestinal microbiota on gut motility and constipation. *Advances in Nutrition: An International Review Journal*.

[20] Martínez-Martínez M. I., Calabuig-Tolsá O., Cauli O. (2017). The effect of probiotics as a treatment for constipation in elderly people: a systematic review. *Archives of Gerontology and Geriatrics*.

[21] Ge X., Ding C., Gong J. (2016). Short-term efficacy on fecal microbiota transplantation combined with soluble dietary fiber andprobiotics in the treatment of slow transit constipation. *Zhonghua Wei Chang Wai Ke Za Zhi*.

[22] Dinning P. G., Fuentealba S. E., Kennedy M. L., Lubowski I. J., Cook I. J. (2007). Sacral nerve stimulation induces pan-colonic propagating pressure waves and increases defecation frequency in patients with slow-transit constipation. *Colorectal Disease*.

[23] Dinning P. G., Hunt L. M., Arkwright J. W. (2012). Pancolonic motor response to subsensory and suprasensory sacral nerve stimulation in patients with slow-transit constipation. *British Journal of Surgery*.

[24] Jin X., Ding Y.-j., Wang L.-l., Shu-ging D., Lin S. (2010). Clinical study on acupuncture for treatment of chronic functional constipation. *Chinese Acupuncture & Moxibustion*.

[25] Hui-fen Z., Shu-qing D., Yi-jiang D., Wang l.-l., Liu H. (2014). Observation on effect characteristics of electroacupuncture for different types of functional constipation. *Chinese Acupuncture & Moxibustion*.

[26] Ni M., Ding S., Shaohua H., Zhou H., Wang L. (2013). Translumbar motor-evoked potentials in diagnosis of functional defecation disorders. *Third Military Medical University*.

[27] Wang L.-l., Jin X. (2014). Re-understanding of eight Liao points. *Journal of Nanjing University of TCM*.

[28] Carrington E. V., Evers J., Grossi U. (2015). A systematic review of sacral nerve stimulation mechanisms in the treatment of fecal incontinence and constipation. *Neurogastroenterology & Motility*.

[29] Wu S., Cheng Y.-R., Zhou J.-Y., Wu B.-S., Chen Yu-G., Yang Bo-L. (2014). Expression of AQP3 and 8 in loperamide induced constipation in rats. *World Chinese Journal of Digestology*.

[30] Huang X., Chen L., Chen C., Zhang L., Liu m. (2015). Comparison of three kinds of type constipation model. *Sichuan Journal of Zoology*.

[31] Yu J., Liu Z., Ma X. (2007). The location and anatomical structure of BL32 of rats. *Acupuncture Research*.

[32] Liu C., Wang Y., Qian M. O. (2013). Electroacupuncture at BL32/BL33 in the inhibition of overactive bladder and its acupoints specificity. *Global Traditional Chinese Medicine*.

[33] Zhou Y., Wang Y., Zhang H., Yan S., Wang B., Xie P. (2016). Effect of vasoactive intestinal peptide on defecation and VIP-cAMP-PKA-AQP3 signaling pathway in rats with constipation. *Zhong Nan Da Xue Xue Bao Yi Xue Ban*.

[34] Devane L. A., Evers J., Jones J. F. (2013). A review of sacral nerve stimulation parameters used in the treatment of faecal incontinence. *Surgeon*.

[35] Ratto C., Ganio G., Naldini G. (2015). Long-term results following sacral nerve stimulation for chronic constipation. *Colorectal Disease*.

[36] Thomas G. P., Vaizey C. J., Dudding T. C. (2015). A double-blinded randomized multicentre study to investigate the effect of changes in stimulation parameters on sacral nerve stimulation for constipation. *Colorectal Disease*.

[37] Liu N. (2012). *The Clinic Observation of Achalasia Pelvic Floor Syndrome by Different Electroacupuncture Frequency*.

[38] Huang Z. (2016). *“ Effects and Mechanism of Sacral Nerve Stimulation on Slow Transit Constipation*.

[39] Worsoe J., Fassov J., Schlageter V., Rijkhoff N. J., Laurberg S., Krogh K. (2012). Turning off sacral nerve stimulation does not affect gastric and small intestinal motility in patients treated for faecal incontinence. *Colorectal Disease: The Official Journal of the Association of Coloproctology of Great Britain and Ireland*.

[40] Shin A., Camilleri M., Erwin P., West C. P., Murad M. H. (2014). Systematic review with meta-analysis: highly selective 5-HT4 agonists (prucalopride, velusetrag or naronapride) in chronic constipation. *Alimentary Pharmacology & Therapeutics*.

[41] Wang Z. (2000). New type of gastrointestinal motility drug mosapride (berluscan). *Chinese Journal of New Drugs and Clinical Remedies*.

[42] Ji S. W., Park H. J., Cho J. S., Lim J. H., Lee S. I., Lee (2003). Investigation into the effects of mosapride on motility of Guinea pig stomach, ileum, and colon. *Yonsei Medical Journal*.

[43] Gershon M. D., Tack J. (2007). The serotonin signaling system: from basic understanding to drug development for functional GI disorders. *Gastroenterology*.

[44] Linthorst A. C. E., Peñalva R. G., Flachskamm C., Holsboer F., Reul J. M. H. M. (2002). Forced swim stress activates rat hippocampal serotonergic neurotransmission involving a corticotropin-releasing hormone receptor-dependent mechanism. *European Journal of Neuroscience*.

[45] Wood J. D. (2011). Enteric nervous system: the brain-in-the-gut. *Colloquium Series on Integrated Systems Physiology: From Molecule to Function*.

[46] Huang Z., Wei Z., Zhang J., Wu X. (2016). The relationship between substance P, vasoactive intestinal peptide and the intestinal tract of mice with spleen deficiency and constipation. *Clincal Journal of Chinese Medicine*.

[47] Yan-Hui S., Sun Y.-H., Zhao Z.-s. (2012). Effects of warming acupuncture on substance-P and vasoactine intrestinal peptide in rats with slow transit constipation. *Chinese Journal of Gerontology*.

[48] Lysy J., Goldin E., Sestieri M. (1997). Decreased substance P content in the rectal mucosa of diabetics with diarrhea and constipation. *Metabolism*.

[49] Zhao B., Kong P., Wu Z., Tang X. (2016). Experimental study on the relationship between 5-HT and slow transit constipation. *Journal of Colorectal Disease*.

[50] Yang L., Zheng S., Xiao-meil W., Jiang Y., Xiao-xu L. (2014). Regulating effect of electroacupuncture on colonic 5-HT and 5-HT4 receptors in rats with irritable bowel syndrome of constipation type. *Shanghai Journal Acu-Mox*.

[51] Costcdio M. M., Coatcs M. D., Brooks E. M (2010). Mu-cosal serotonin signaling is altered in chronic constipation but not in opiate-induced constipation. *The American Journal of Gastroenterology*.

[52] Chen B., Ming-yue L. I., Zhao X., Yang-yang L., Guo Yi (2014). Advances in studies on substance P and acupuncture effect. *Shanghai Journal Acu-Mox*.

[53] Koppen I. J., Benninga M. A., Tabbers M. M. (2016). Is there A role for pre-, pro- and synbiotics in the treatment of functional constipation in children? A systematic review. *Journal of Pediatric Gastroenterology & Nutrition*.

[54] Lian L., Liu C. (2017). Review on intestinal flora and functional consipation. *Liaoning Journal of Traditional Chinese Medicine”*.

[55] Gen-lin L., Zhang Y.-y., Li H.-b. (2016). Effect of constipation induced by diphenoxylate on intestinal flora in a rat. *Chinese Journal of Tissue Engineering Research*.

[56] Shang-kui X., Ren D.-l., Zheng-yu X., Peng h., Xiao-xue W. (2016). Changes of intestinal flora and effects of corresponding intervention in colon slow transit constipation rats. *Guangdong Medical Journal*.

[57] Huang L., Gao R., Yan X., Zhu Q., Cheng P. (2017). Structure analysis of the gut microbiota in chronic functional constipation patients. *Journal of Colorectal Disease (Electronic Edition)*.

[58] Tim G. J., Meij d., FJ de Groot E., Eck A. (2016). Characterization of microbiota in children with chronic FunctionalConstipation. *PLoS One*.

[59] Parthasarathy G., Chen J., Chen X. (2016). Relationship between microbiota of the colonic mucosa vs feces and symptoms, colonic transit, and methane production in female patients with chronic constipation. *Gastroenterology*.

[60] Magnus S., Giovanni B., Flint H. J. (2013). Intestinal microbiota in functional bowel disorders: a Rome foundation report. *Gut*.

[61] Agrawal A., Houghton L. A., Morris J. (2009). Clinical trial: the effects of a fermented milk product containing Bifidobacterium lactis DN-173010 on abdominal distension and gastrointestinal transit in irritable bowel syndrome with constipation. *Alimentary Pharmacology & Therapeutics*.

[62] Wang X.-l., Wang W.-h., Dai Y., Wang C., Xin-hong D. (2014). Probiotics/prebiotics preparation for functional constipation: a systemic review and meta-analysis. *Clinical Medication Journal*.

[63] Chen R. (2008). The basic characteristics and time-dependent effects of acupuncture and Moxibustion. *Journal of Jiangxi University of TCM*.

